# TcMAC21 mouse model recapitulates abnormal vascular physiology observed in humans with Down syndrome

**DOI:** 10.14814/phy2.70384

**Published:** 2025-06-06

**Authors:** Xiangyu Zheng, Mostafa Sabouri, Bryan J. Irwin, Joseph Bernardo, Daniel R. Machin

**Affiliations:** ^1^ Department of Nutrition and Integrative Physiology Florida State University Tallahassee Florida USA; ^2^ Cardiology Division, Department of Medicine Emory University School of Medicine Atlanta Georgia USA; ^3^ Vascular Physiology Group, Department of Cell Biology and Physiology University of New Mexico School of Medicine Albuquerque New Mexico USA; ^4^ Department of Biological Science Florida State University Tallahassee Florida USA

**Keywords:** aortic stiffness, blood pressure, endothelium, glycocalyx, trisomy 21

## Abstract

People with Down syndrome (DS) have abnormal vascular physiology, demonstrated by low systolic blood pressure (BP) and low aortic stiffness that are accompanied by endothelial dysfunction. The TcMAC21 mouse model of DS has many features observed in people with DS, although vascular physiology has not been studied. At 4 months old, male and female TcMAC21 mice exhibited lower systolic BP and aortic stiffness, as determined by aortic pulse wave velocity, which are accompanied by blunted carotid artery flow‐mediated vasodilation, indicating endothelial dysfunction, compared to euploid (i.e., control) mice. To determine a potential mechanism for blunted flow‐mediated vasodilation, we assessed endothelial glycocalyx properties, which mechanotransduces fluid shear stress to the endothelial cells, stimulating flow‐mediated vasodilation. We observed a lower glycocalyx thickness in the mesenteric microcirculation of TcMAC21 mice. Vascular abnormalities in TcMAC21 mice were accompanied by systemic inflammation. This is the first study to examine vascular physiology in the TcMAC21 mouse model of DS and investigate glycocalyx properties in any model of DS, including humans. Taken together, these findings support the use of the TcMAC21 mouse model to study the vascular physiology in people with DS and may provide translational insight into the role of glycocalyx in vascular abnormalities in DS.

## 1 INTRODUCTION

Down syndrome (DS) is a genetic disorder that results from the triplication of human chromosome 21 (HSA21) (Stallings et al., 2024). The prevalence of DS is ~1 in every 600 live births, making it the most common genetic disorder in the United States (Stallings et al., 2024). The population size and life expectancy for people with DS have been expanding over the past several decades (de Graaf et al., 2017; Presson et al., 2013), mainly due to the treatment of congenital heart disease in infancy, which affects ~50% of people with DS (Bates et al., 2023; Landes et al., 2020; Roizen et al., 2014). Still, cardiovascular diseases (CVDs) remain the leading cause of death in people with DS (Englund et al., 2013; Zhu et al., 2013), as people with DS are at higher risk for several cardiovascular complications, such as coronary and cerebral artery diseases (Dimopoulos et al., 2023).

A central feature of CVD in the general population is vascular dysfunction, such as elevated systolic blood pressure (BP), aortic stiffening, and endothelial dysfunction (Lakatta & Levy, 2003), which all contribute to the CVD development (Fuchs & Whelton, 2020; Mitchell et al., 2010; Widmer & Lerman, 2014). While people with DS are at higher risk for CVD, they exhibit abnormal vascular physiology with lower systolic BP and aortic stiffness (Rodrigues et al., 2011), but paradoxically have endothelial dysfunction compared to people without DS (Cappelli‐Bigazzi et al., 2004). In recent years, there is emerging evidence that endothelial dysfunction may be initiated by a dysfunctional endothelial glycocalyx (Feihl et al., 2006; Noble et al., 2008; Ushiyama et al., 2016). The endothelial glycocalyx is a negatively charged gel‐like structure that is bound to the luminal surface of the endothelium and acts as a barrier to flowing blood (Pries et al., 2000; van den Berg et al., 2006; Yao et al., 2007). Indeed, there are several CVD states that exhibit vascular dysfunction that are also accompanied by a degraded glycocalyx (Frech et al., 2015; Gonzalez & Selwyn, 2003; Hamdy et al., 2003; Machin et al., 2018; Yilmaz et al., 2006). From a functional standpoint, the glycocalyx directly participates in vasodilation by mechanotransducing fluid shear stress from flow to the endothelium, which stimulates nitric oxide (NO)‐mediated vasodilation (Pries et al., 2000; Yao et al., 2007). Although it is possible that a degraded glycocalyx may impair endothelial function in DS, currently, no studies in people with DS or preclinical models of DS have examined any aspects of glycocalyx properties.

The Ts65dn mouse, first characterized in 1993, is the most popular mouse model of DS (Davisson et al., 1993). However, there are limitations to this model, as only ~52% of HSA21 protein‐coding genes are present in the Ts65dn mouse, which may explain why some interventions that have shown success in Ts65dn mice have not been translated to humans (Guedj et al., 2023). Recently, the TcMAC21 mouse model of DS was developed, which is the most complete genetic model of DS available, incorporating an artificial mouse chromosome that contains ~93% of HSA21 protein‐coding genes (Kazuki et al., 2020). The TcMAC21 mouse recapitulates many DS phenotypes, including congenital heart anomalies, developmental delays, and cognitive impairments (Kazuki et al., 2020). However, the vascular physiology in these mice has not been studied. Therefore, in this study, we sought to examine the vascular physiology of TcMAC21 mice, including glycocalyx properties. We hypothesized that TcMAC21 mice would demonstrate an abnormal vascular physiology that was similar to people with DS.

## 2 METHODS

### 2.1 Ethical approval

All animal procedures conform to the *Guide to the Care and Use of Laboratory Animals: Eighth Edition* (National Research Council (U.S.) et al., 2011) and were approved by the Florida State University Animal Care and Use Committee.

### 2.2 Animals

**FIGURE 1:**
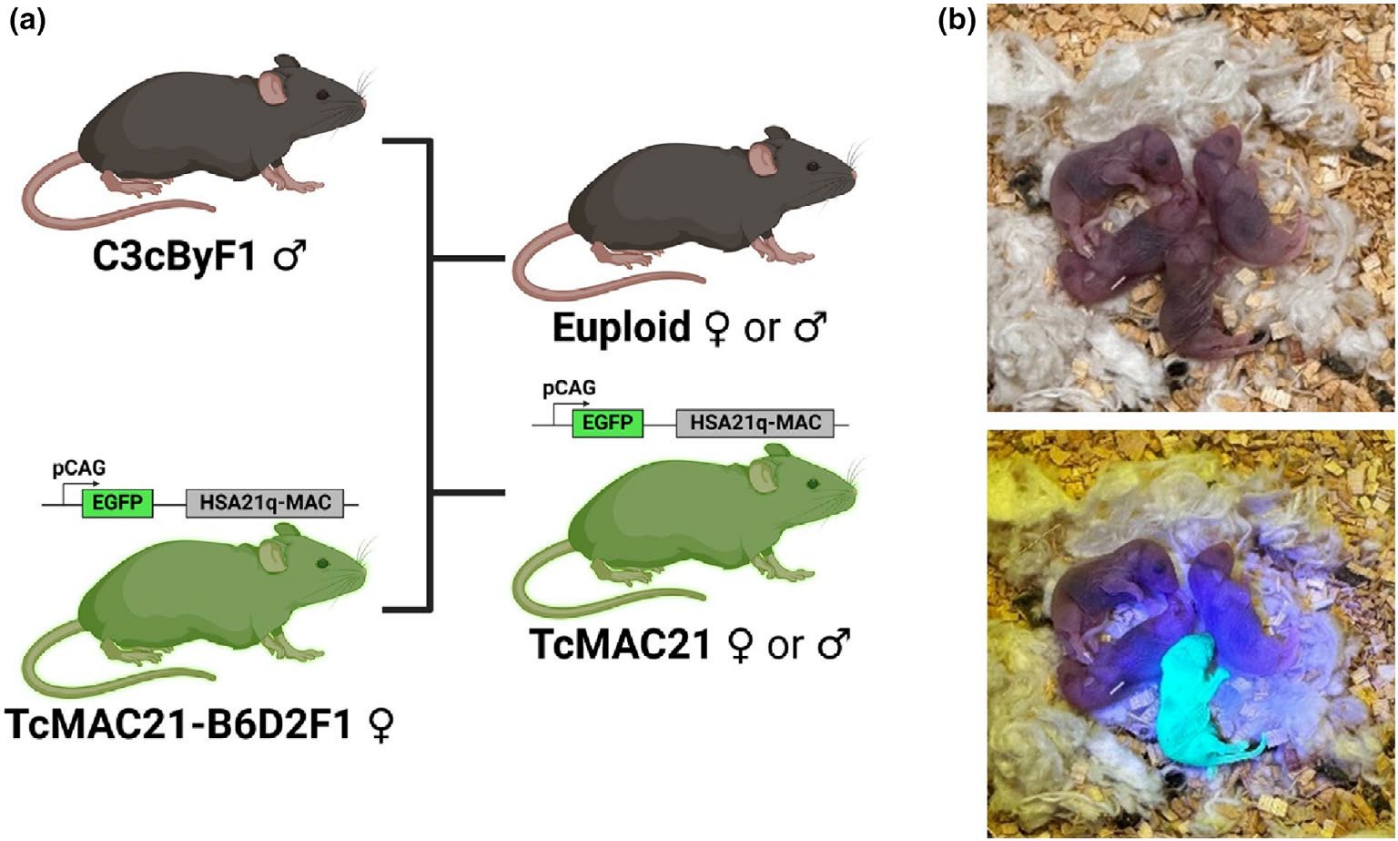
Breeding scheme of TcMAC21 and euploid mice (a) and identification of GFP‐positive, TcMAC21 and GFP‐negative, euploid mice (b).

Male and female TcMAC21 and euploid (i.e., control) mice used in this study were first generation offspring of male C3cByF1 and female TcMAC21‐B6D2F1 mice (Figure 1a). Male TcMAC21 mice are not fertile, thus, female TcMAC21 mice on a B6D2F1 background were purchased from the Jackson Laboratory to be used as breeders (Kazuki et al., 2020). Male C3cByF1 breeders were generated from male BALB/cByJ and female C3H/HeJ mice that were purchased from Jackson Laboratory. The breeding scheme was designed to increase genetic diversity by creating a mouse crossed from four different inbred strains similar to the outbred, UM‐HET strain of mice (Miller & Chrisp, 1999). All mice were housed in standard mouse cages under a 12:12 light: dark cycle in a temperature‐controlled environment. Mice were fed standard rodent chow (LabDiet No. 5001). Food and water were supplied *ad libitum*. Euploid TcMAC21 (Here on “Euploid”) or trisomy TcMAC21 (Here on “TcMAC21”) mice were identified by illuminating with UV light (Xite™ Fluorescence Flashlight System, EMS, Hatfield, PA) as TcMAC21 but not euploid mice contain peri‐centromeric GFP on the mouse artificial HSA21 (Figure 1b) (Kazuki et al., 2020).

### 2.3 Systolic blood pressure

Arterial systolic BP was determined in vivo in conscious mice using the tail‐cuff method (MC4000 BP analysis system; Hatteras Instrument, Cary, NC, USA) at 4 months old, as described previously (Trott et al., 2014). This method has been validated versus arterial catheter BP measurement (Feng et al., 2008). Briefly, mice underwent five consecutive days of tail cuff BP measurement that were conducted in a quiet and warm (~23°C) environment at the same time of the day (Bastianini et al., 2012). During each trial, mice were restrained on a heated platform (40°C). Each trial consisted of five preliminary measurements that were followed by 10 experimental measurements. Measurements with aberrant movement/behavior or poor signal were excluded. If more than five values were excluded, the measurement was repeated. Values from all experimental measurements were used to calculate mean values for each mouse.

### 2.4 Aortic stiffness

Aortic stiffness was assessed by aortic pulse wave velocity (PWV) measurement in vivo at 4 months old, as described previously (Machin et al., 2020). Briefly, mice were anesthetized with isoflurane (3%) in room air at 100 mL/min flow rate and placed in the supine position on a heated platform (37°C). Blood velocity waveforms at the transverse aortic arch and at the abdominal aorta were obtained simultaneously with two 20‐MHz Doppler probes (Indus Instruments, Webster, TX, USA) and recorded using PowerLab 16/35 with LabChart 8 software (AD Instruments Inc., Colorado Springs, CO, USA) at a sampling rate of 100 k/s. After blood velocity waveforms were collected, a precise measurement of the traveled distance between the Doppler probes was recorded using a scientific caliper. The transit time between Doppler sites was determined using the foot‐to‐foot method with LabChart Lightening 1.8 software (AD Instruments Inc., Colorado Springs, CO, USA). Aortic PWV was calculated as the traveled distance divided by the transit time.

### 2.5 Aortic histology

A 2–3 mm aortic ring with perivascular tissue intact was excised from the thoracic aorta and embedded in Optimal Cutting Temperature medium, as described previously (Machin et al., 2020). Aortic rings were sliced into 8‐micron sections. Each slide contained two to three aortic sections, which were averaged. For measures of medial cross‐sectional area (CSA), the lumen border and the outer medial border were traced in ImageJ, and internal areas were measured. These areas were used to calculate medial CSA and were calculated as the outer media border area minus the lumen area. Collagen was quantified by Masson's trichrome staining (MilliporeSigma, Burlington, MA, USA) as a percentage of the selected area, as described previously (Machin et al., 2023; Walker et al., 2019). Blue channel images from an RGB stack were used for densitometric quantification of collagen content with ImageJ. Elastin was quantified by Verhoff‐Van Gieson staining (Abcam, Cambridge, UK), as described previously (Machin et al., 2020). An 8‐bit grayscale was used for densitometric quantification of elastin content with ImageJ. Collagen and elastin content were normalized to euploid mice by dividing individual values by the means of euploid mice for each measurement.

### 2.6 Ex vivo arterial function

To assess ex vivo arterial function, both carotid arteries were excised, cleared of surrounding tissue, cannulated in the stage of a pressure myograph (DMT Inc., Hinnerup, Denmark), and perfused with physiological salt solution that contains 145.0 mM NaCl, 4.7 mM KCl, 2.0 mM CaCl_2_, 1.17 mM MgSO_4_, 1.2 mM NaH_2_PO_4_, 5.0 mM glucose, 2.0 mM pyruvate, 0.02 mM EDTA, 3.0 mM MOPS buffer, and 0.5% BSA, and pH 7.4 at 37°C. First, vasocontractility was assessed in response to the cumulative addition of phenylephrine (P6126, Sigma‐Aldrich; 1 × 10^−10^ to 1 × 10^−4^ M) in these arteries. Then, arteries were submaximally pre‐constricted with 2 μM phenylephrine and endothelium‐dependent dilation (EDD) was measured in response to incremental intraluminal flow by creating a pressure gradient (Δ4, Δ10, Δ20 cm H_2_O) or the cumulative addition of acetylcholine (A6625, Sigma‐Aldrich; 1 × 10^−10^ to 1 × 10^−4^ M), respectively, in the absence or presence of the NO synthase inhibitor, L‐NAME (AC328750050, Acros Organics; 0.1 mM, 30 min), as described previously (Caldwell et al., 2021; Zheng et al., 2023). Endothelium‐independent dilation (EID) was assessed in response to the cumulative addition of sodium nitroprusside (AC424360250, Acros Organics; 1 × 10^−10^ to 1 × 10^−4^ M). Following ex vivo arterial function measurements, carotid arteries were incubated in Ca^2+^‐free physiological salt solution for 1 h to determine their maximal diameter. Luminal diameters were measured by VasoTracker software 2 min after each change in bath concentration/pressure gradient (Lawton et al., 2019).

### 2.7 Intravital microscopy

The mesenteric microcirculation was observed using intravital microscopy in mice anesthetized with isoflurane (3%) in room air at 100 mL/min flow rate, as described previously (Zheng et al., 2023; Zheng, Deacon, et al., 2022). Intravital microscopy was performed with a handheld video capillary microscope (CapiScope HVCS, KK Technology, Honiton, UK) to view the mesenteric microcirculation. Video of the mesenteric microcirculation was recorded and analyzed using an automated capture and analysis system (GlycoCheck, MicroVascular Health Solutions LLC, Alpine, UT). In each mouse, 24 trials were acquired with each trial consisting of several 2‐s video recordings that capture ~5000 microvessel segments that are 10 μm in length and range from 4 to 25 μm in perfused diameter. After 2–3 trials were obtained in one location, the microscope was moved to a different location to counterbalance the spatial heterogeneity within the mesenteric microcirculation.

The automated analysis algorithm identifies perfused microvessel segments as any segment with sufficient contrast that contains red blood cells (RBCs) in ≥50% of its length in the first frame of a video recording. Microvascular density and capillary blood volume (CBV) are calculated using the following equation:

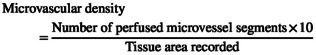




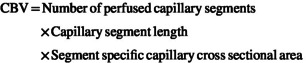




RBC velocities are determined by dividing RBC displacement by the time between consecutive video frames, and microvascular blood flow was determined in 9–11 μm diameter microvessels, as described previously (Rovas et al., 2021).

Perfused boundary region (PBR) represents the depth of penetration of RBCs into the endothelial glycocalyx and is taken as a marker of glycocalyx barrier function, with a larger PBR indicating greater perturbation of the glycocalyx. PBR is calculated using the following equation:

$$\mathrm{PBR}=\frac{\mathrm{RBC}‐\mathrm{Perfused}\;\mathrm{vessel}\;\mathrm{diameter}-\text{Median}\;\mathrm{RBC}\;\text{column width}}{2}$$

Manual determination of glycocalyx thickness was performed by measuring the change in width of flowing RBC column before and after the passage of a spontaneous white blood cell (WBC) in individual capillaries, as described previously (Nieuwdorp et al., 2008).

### 2.8 Metabolic testing

Metabolic function was determined using the glucose tolerance test (GTT) and insulin tolerance test (ITT), as described previously (Zheng, Li, et al., 2022). Briefly, mice were fasted for 4 h in the morning at the same time of the day. Baseline blood glucose was measured using a glucometer (Clarity BG1000; Clarity Diagnostics, Boca Raton, FL) in blood collected via a tail cut. Mice were injected intraperitoneally with glucose (2 g/kg body mass) or insulin (1 U/kg body mass). Blood glucose was assessed at 15, 30, 45, 60, 90, and 120 min after injection.

### 2.9 Blood analysis

During euthanasia, blood was collected via cardiac puncture using heparinized needles. Immediately following collection, whole blood was analyzed using a hematological analyzer (Heska Element HT5, Loveland, CO) for differential cell counts, as described previously (Laitano et al., 2021). Immediately after, whole blood was centrifuged at 4°C for 20 min at 3000*g*. Plasma was aliquoted and stored at −80°C. Plasma cytokines/chemokines were measured using multiplex panels (MCYTMAG‐70K‐PX32, Millipore Sigma, Burlington, MA), as described previously (Renteria et al., 2023).

### 2.10 Statistical analysis

Statistical tests were performed with GraphPad Prism 10 (GraphPad Software Inc., San Diego, CA). An unpaired *t*‐test was used to evaluate differences between groups in data sets that passed Shapiro–Wilk normality test. Otherwise, a Mann–Whitney *U* test was used to evaluate differences between groups. A two‐way, mixed model ANOVA with Holm‐Šídák post hoc test was used to evaluate differences between groups. Bivariate correlational analysis was determined between selected variables. Significance level α was set at 0.05 for all statistical tests. Data are presented as means ± SEM.

## 3 RESULTS

### 3.1 Animal characteristics

Animal characteristics are presented in Table 1. TcMAC21 and euploid mice were similar age at sacrifice (*p* > 0.05). TcMAC21 had lower body mass and tibia length than euploid mice (*p* < 0.05 for both). For tissue masses, TcMAC21 had lower heart, liver, quadricep, gastrocnemius, plantaris, kidney, visceral adipose tissue, and subcutaneous adipose tissue mass compared to euploid mice (*p* < 0.05 for all), but spleen mass was similar (*p* > 0.05), and TcMAC21 had higher soleus mass compared to euploid mice (*p* < 0.05). For tissue masses when normalized to body mass, TcMAC21 had lower tissue/body mass of heart, liver, quadricep, gastrocnemius, plantaris, and kidney compared to euploid mice (Table S1; *p* < 0.05 for all), but there was a similar tissue/body mass of spleen, visceral adipose tissue, and subcutaneous adipose tissue (*p* > 0.05 for all), and TcMAC21 had higher tissue/body mass of soleus compared to euploid mice (*p* < 0.05). For tissue masses when normalized to tibia length, TcMAC21 had lower tissue mass/tibia length of heart, liver, quadricep, gastrocnemius, plantaris, and kidney compared to euploid mice (*p* < 0.05 for all), but there was a similar tissue mass/tibia length of spleen, visceral adipose tissue, and subcutaneous adipose tissue (*p* > 0.05 for all), and TcMAC21 had higher tissue mass/tibia length of soleus compared to euploid mice (*p* < 0.05). There were also sex‐specific differences in animal characteristics that are presented in Table S2.

**TABLE 1:** Animal characteristics.

	Euploid	TcMAC21	Group
Male/Female^#^	**11/11**	**12/7**	
Age, mo	4.3 ± 0.5	4.4 ± 0.3	0.3474
Body mass, g	28.2 ± 3.8	21.2 ± 3.8	<0.0001
Tibia length, mm	18.0 ± 0.5	17.6 ± 0.7	0.0440
Heart, mg	126 ± 17	108 ± 16	0.0016
Liver, mg	1361 ± 165	1227 ± 171	0.0160
Spleen, mg	83 ± 19	72 ± 14	0.0543
Quadricep, mg	191 ± 30	93 ± 19	<0.0001
Gastrocnemius, mg	132 ± 20	64 ± 17	<0.0001
Soleus, mg	7 ± 2	9 ± 2	0.0397
Plantaris, mg	19 ± 4	12 ± 3	<0.0001
Kidney, mg	203 ± 33	173 ± 33	0.0059
VAT, mg	418 ± 342	218 ± 116	0.0157
SAT, mg	195 ± 97	124 ± 43	0.0047

*Note*: Group comparisons in euploid and TcMAC21 mice were analyzed using unpaired *t*‐test or Mann–Whitney *U* test to identify differences in animal characteristics. Data are individual values and means ± SD.

Abbreviations: VAT, visceral adipose tissue; SAT, subcutaneous adipose tissue.

^#^ Bold values indicates male and female TcMAC21 and euploid.

### 3.2 Systolic blood pressure and aortic function and structure

**FIGURE 2:**
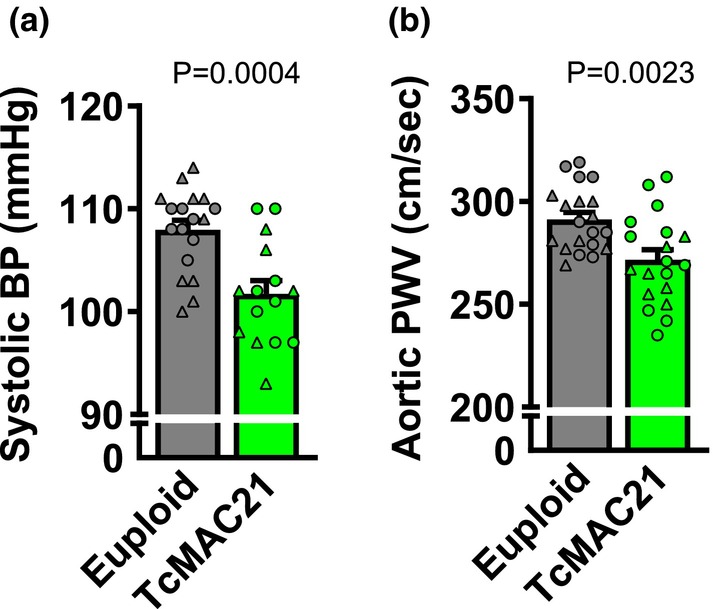
Group comparisons in euploid and TcMAC21 mice were analyzed using unpaired *t*‐test to identify differences in systolic blood pressure (BP; a; *n* = 15–18 mice/group) and aortic pulse wave velocity (PWV; b; *n* = 19–20 mice/group). *p < 0.05 versus euploid. Data are individual values (males = circles, females = triangles) and means±SEM.

**FIGURE 3:**
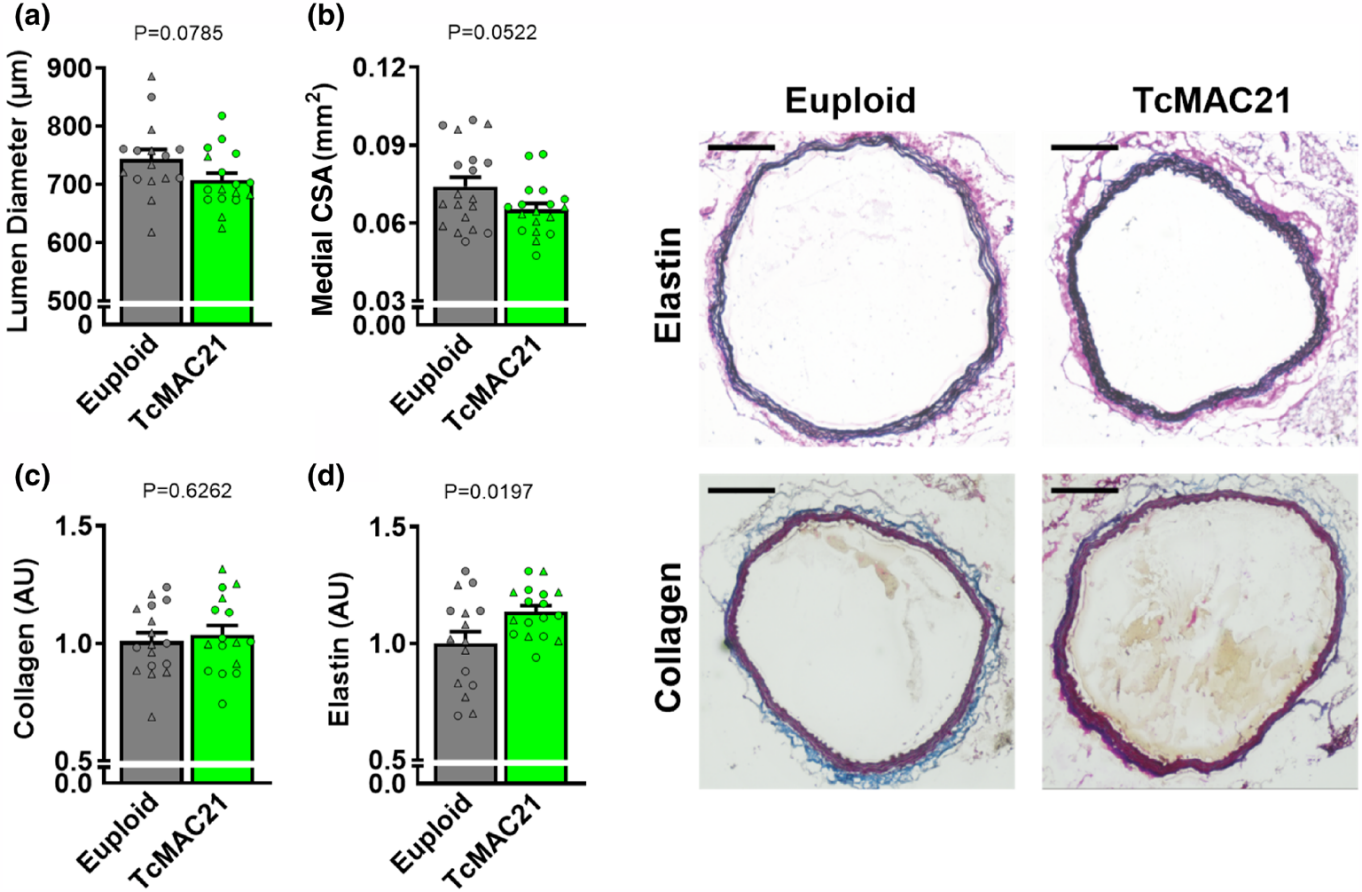
Group comparisons in euploid and TcMAC21 mice were analyzed using unpaired *t*‐test to identify differences in aortic lumen diameter (a; *n* = 16–18 mice/group), aortic medial cross‐sectional area (b; *n* = 18–19 mice/group), elastin content (c; *n* = 14–16 mice/group), and collagen content (d; *n* = 16–17 mice/group). Images are accompanied by representative images of collagen and elastin staining. Black scale bars are equal to 200 μm. *p < 0.05 versus euploid. Data are individual values (males = circles, females = triangles) and means ± SEM.

We observed a lower systolic BP in TcMAC21 compared to euploid mice (Figure 2a; *p* < 0.05). Similarly, TcMAC21 had a lower aortic PWV compared to euploid mice (Figure 2b; *p* < 0.05). In histological sections of thoracic aortas, we observed similar lumen diameter and medial CSA between groups (Figure 3a,b; *p* > 0.05 for both). While collagen content was similar between groups (Figure 3c; *p* > 0.05), TcMAC21 had a higher elastin content in the aorta compared to euploid mice (Figure 3d; *p* < 0.05). There was no relationship between aortic PWV and collagen (*r* ^2^ = 0.00) or elastin content (*r* ^2^ = 0.00) (Figure S1a,b; *p* > 0.05).

### 3.3 Ex vivo arterial function

**FIGURE 4:**
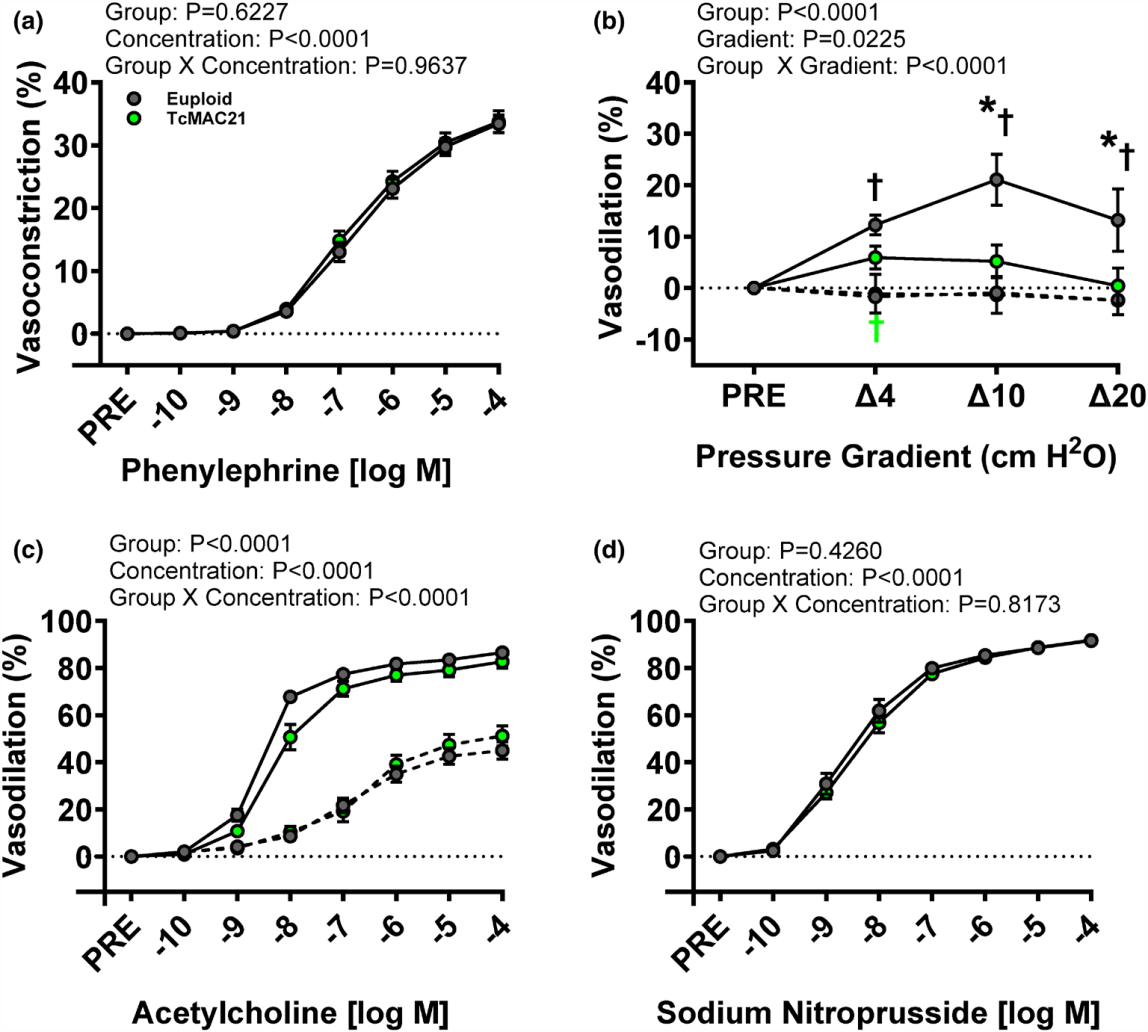
Group × Gradient/Concentration comparisons in euploid and TcMAC21 mice were analyzed using two‐way, mixed model ANOVA with Holm‐Šídák post hoc test to identify differences in carotid artery vasoconstriction to phenylephrine (a; *n* = 19–21 mice/group), as well as vasodilation to changes in pressure gradient in the absence (solid line) and presence of L‐NAME (dashed line) (b; *n* = 9–14 mice/group), acetylcholine in absence (solid line) and presence of L‐NAME (dashed line) (c; *n* = 17–20 mice/group), and sodium nitroprusside (d; *n* = 15 mice/group). *p < 0.05 versus euploid. †*p* < 0.05 versus L‐NAME within the group. Data are means ± SEM.

Ex vivo arterial functional outcomes were assessed in the carotid arteries. The average pre‐constriction percentage was 36.1 ± 1.1% and 38.7 ± 1.4% for carotid arteries from TcMAC21 and euploid mice, respectively (*p* > 0.05). We observed no interaction effect of Group × Concentration on vasocontractility, as assessed by vasoconstriction to phenylephrine (Figure 4a; *p* > 0.05). There was no main effect of Group on vasocontractility (*p* > 0.05), but a significant main effect of Concentration (*p* < 0.05). For EDD, we observed a significant interaction effect of Group × Concentration as well as significant main effects of Group and Concentration with flow‐mediated vasodilation, as assessed by vasodilation to intraluminal flow (Figure 4b; *p* < 0.05 for all). TcMAC21 had lower flow‐mediated vasodilation compared to euploid mice (*p* < 0.05). In the presence of L‐NAME, flow‐mediated vasodilation was lower in the group with L‐NAME compared to groups without L‐NAME, respectively (*p* < 0.05 for both), but similar between groups with L‐NAME (*p* > 0.05). Furthermore, we observed a significant interaction effect of Group × Concentration as well as significant main effects of Group and Concentration on acetylcholine‐mediated vasodilation to acetylcholine (Figure 4c; *p* < 0.05 for all). There was a similar acetylcholine‐mediated vasodilation between TcMAC21 and euploid mice (*p* > 0.05). In the presence of L‐NAME, acetylcholine‐mediated vasodilation was lower in the group with L‐NAME compared to groups without L‐NAME, respectively (*p* < 0.05 for both), but similar between groups with L‐NAME (*p* > 0.05). We observed no interaction effect of Group × Concentration nor main effect of Group on EID, as assessed by vasodilation to sodium nitroprusside (Figure 4d; *p* > 0.05 for both), but there was a significant main effect of Concentration (*p* < 0.05).

### 3.4 Glycocalyx and microcirculatory properties

**FIGURE 5:**
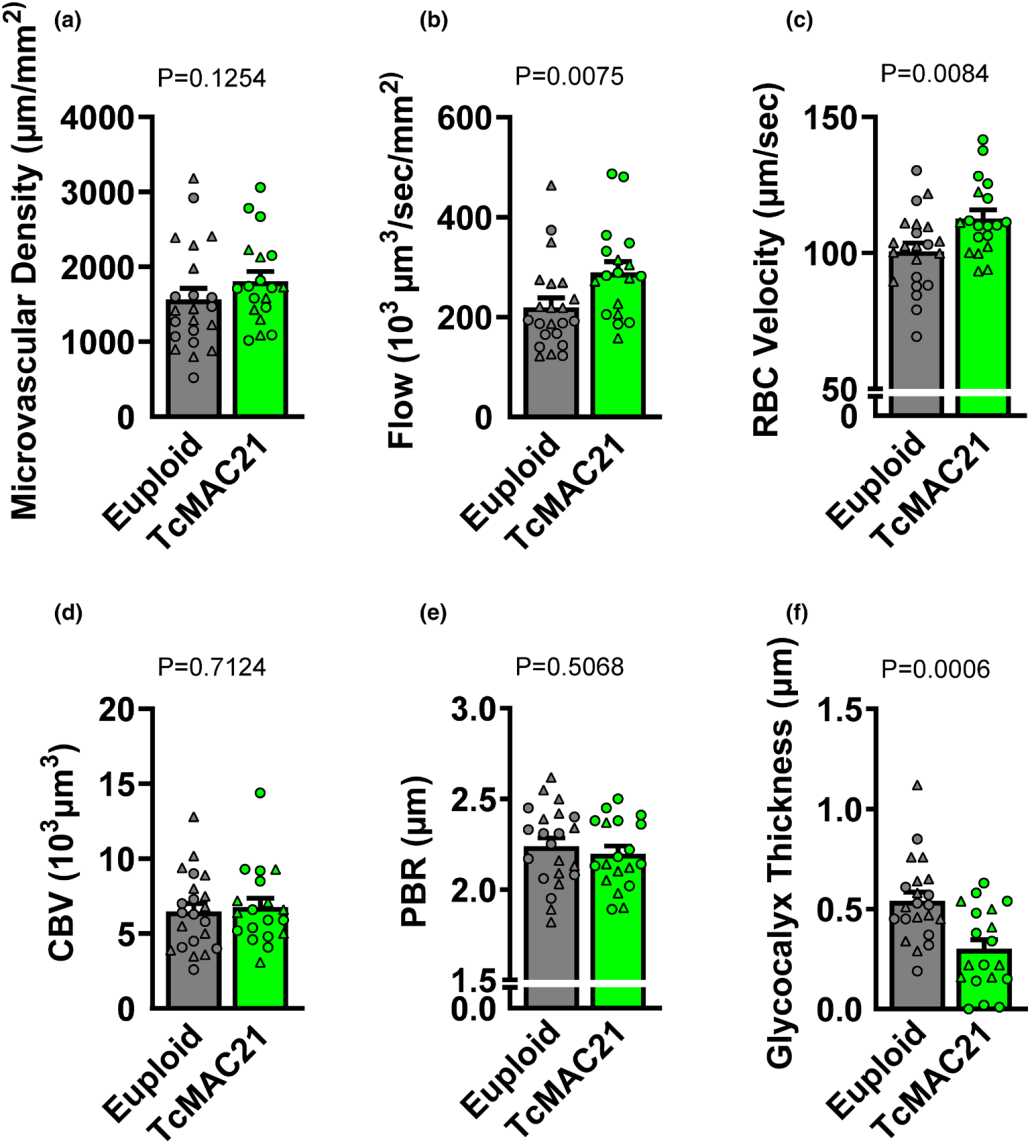
Group comparisons in euploid and TcMAC21 mice were analyzed using unpaired *t*‐test or Mann–Whitney *U* test to identify differences in microvascular density (a; *n* = 19–22 mice/group), flow (b; *n* = 18–22 mice/group), red blood cell (RBC) velocity (c; *n* = 19–22 mice/group), capillary blood volume (CBV; d; *n* = 19–22 mice/group), perfused boundary region (PBR; e; *n* = 19–22 mice/group), and glycocalyx thickness (f; *n* = 19–22 mice/group), . Data are individual values (males = circles, females = triangles) and means ± SEM.

Glycocalyx and microcirculatory properties were assessed in mesenteric microvasculature. We observed similar microvascular density between groups (Figure 5a; *p* > 0.05). Microvascular flow was higher in TcMAC21 mice (Figure 5b; *p* < 0.05). In contrast, RBC velocity was higher in TcMAC21 compared to euploid mice (Figure 5c; *p* < 0.05). CBV was similar between groups (Figure 5d; *p* > 0.05). Lastly, we observed similar PBR between groups (Figure 5e; *p* > 0.05), but TcMAC21 had a lower glycocalyx thickness compared to euploid mice (Figure 5f; *p* < 0.05).

### 3.5 Metabolic function

**FIGURE 6:**
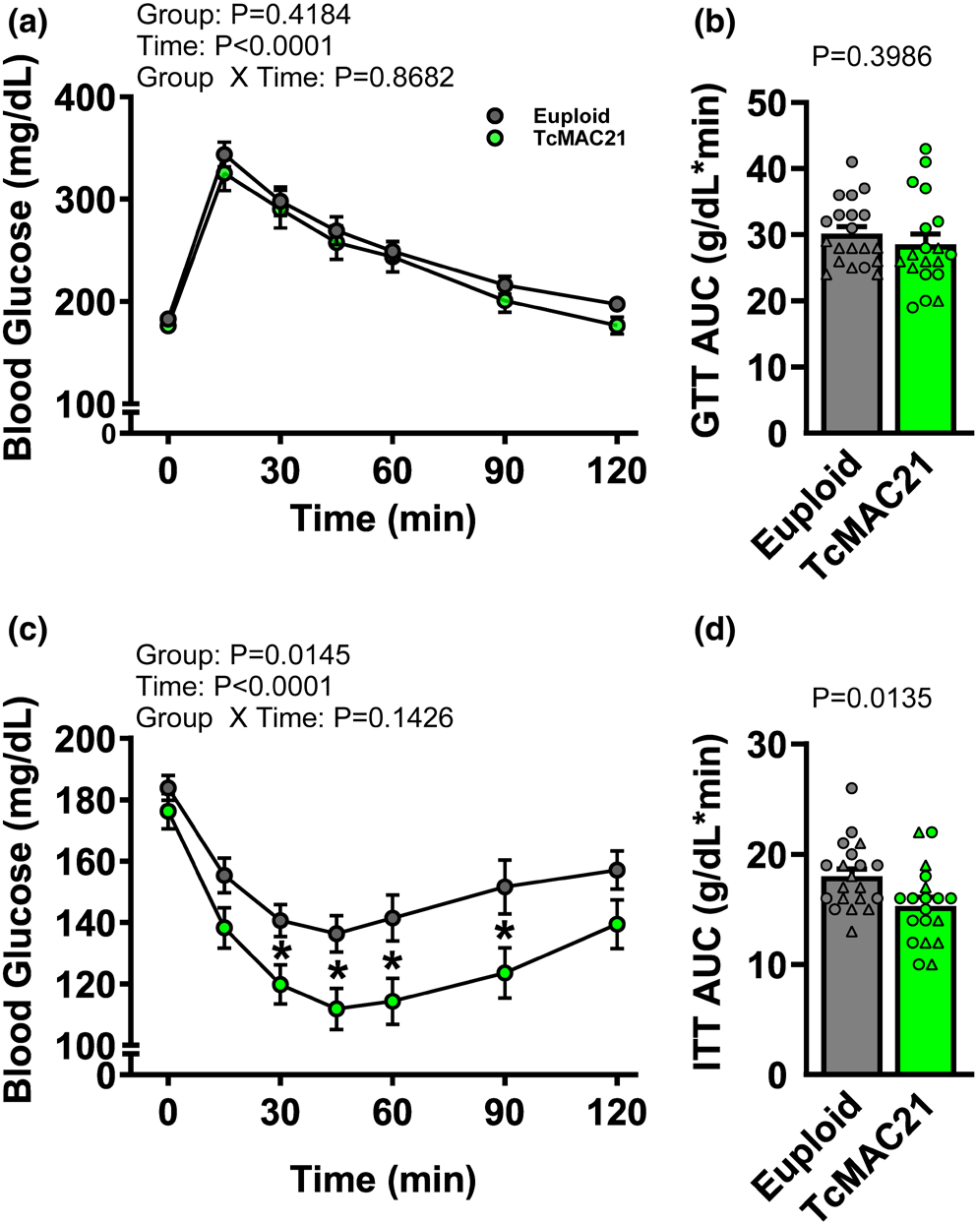
Group × Time comparisons in euploid and TcMAC21 mice were analyzed using two‐way, mixed model ANOVA with Holm‐Šídák post hoc test to identify differences in blood glucose in response to glucose tolerance test (GTT: a; *n* = 19–20 mice/group). An unpaired *t*‐test was used to identify differences in GTT area under the curve (AUC) between groups (b; *n* = 19–20 mice/group). A two‐way, mixed model ANOVA with Holm‐Šídák post hoc test was used to identify differences in blood glucose in response to insulin tolerance test (ITT; c; *n* = 19–20 mice/group; *n* = 19–20 mice/group). An unpaired *t*‐test was used to identify differences in ITT AUC between groups (d). *p < 0.05 versus euploid. Data are individual values (males = circles, females = triangles) and means ± SEM.

We observed no interaction effect of Group × Time on GTT response (Figure 6a; *p* > 0.05). There was no main effect of Group on GTT response (*p* > 0.05), but a significant main effect of Time (*p* < 0.05). There was a similar GTT area under the curve (AUC) between the groups (Figure 6b; *p* > 0.05). We observed no interaction effect of Group × Time on ITT response (Figure 6c; *p* > 0.05). There was significant main effects of Group and Time on ITT response (*p* < 0.05 for both). Specifically, TcMAC21 had lower blood glucose levels at 30‐, 45‐, 60‐, and 90‐min following insulin injection (*p* < 0.05 for all), and lower ITT AUC compared to euploid mice (Figure 6d; *p* < 0.05).

### 3.6 Blood cells and cytokines/chemokines

**FIGURE 7:**
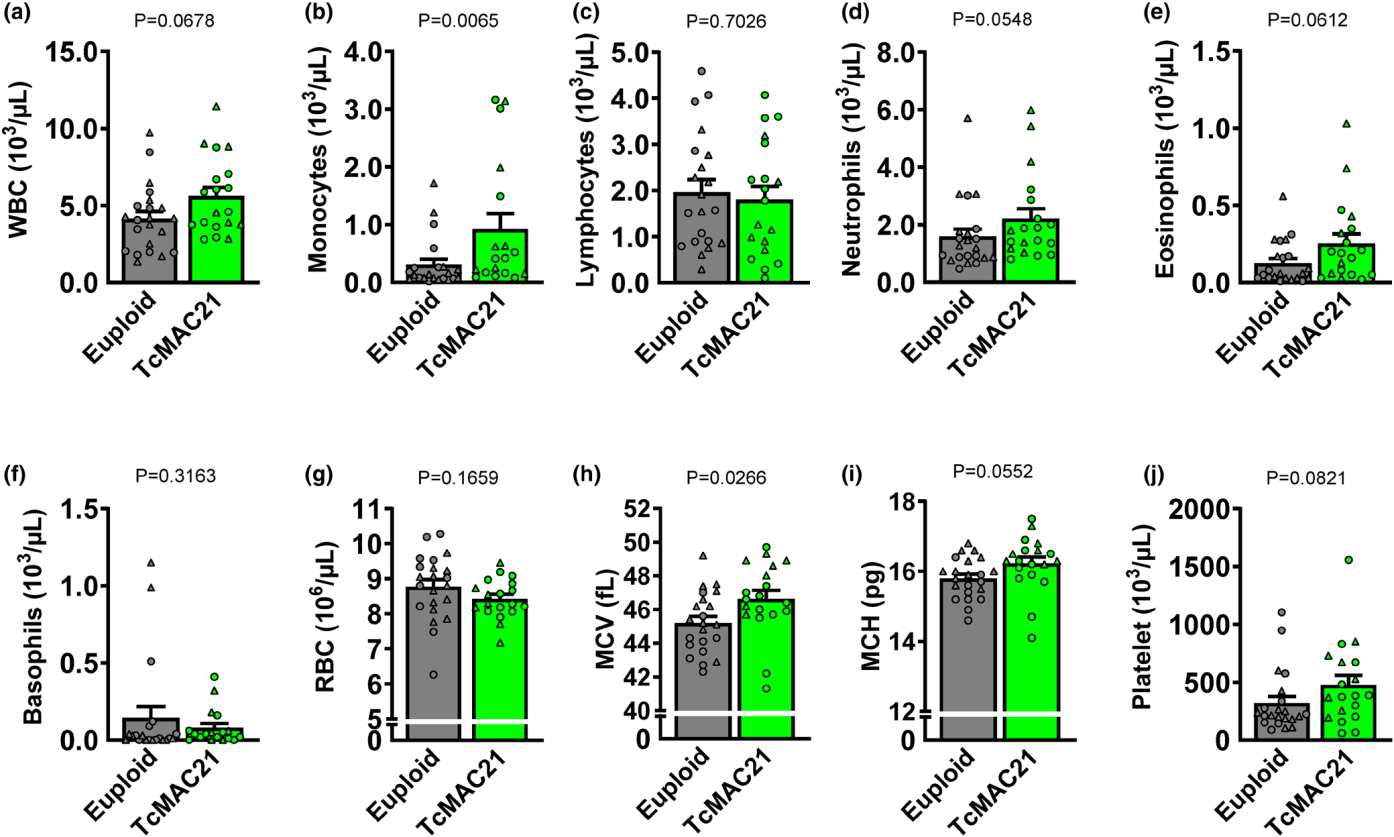
Group comparisons in euploid and TcMAC21 mice were analyzed using unpaired *t*‐test or Mann–Whitney *U* test to identify differences in blood concentrations of white blood cells (WBC; a; *n* = 19–22 mice/group), monocytes (b; *n* = 18–22 mice/group), lymphocytes (c; *n* = 19–22 mice/group), neutrophils (d; *n* = 19–22 mice/group), eosinophils (e; *n* = 19–22 mice/group), basophils (f; *n* = 18–21 mice/group), red blood cells (RBC; g; *n* = 19–22 mice/group), mean corpuscular volume (MCV; h; *n* = 19–22 mice/group), mean corpuscular hemoglobin (MCH; i; *n* = 19–22 mice/group), and platelet (j; *n* = 19–22 mice/group). Data are individual values (males = circles, females = triangles) and means ± SEM.

Although TcMAC21 had similar WBC counts compared to euploid mice (Figure 7a; *p* < 0.05), there was a higher monocyte count compared to euploid mice (Figure 7b; *p* < 0.05), but their lymphocyte, neutrophil, eosinophil, or basophil counts were similar between groups (Figure 7c–f; *p* > 0.05 for all). Although RBC counts between groups were similar (Figure 7g; *p* > 0.05), TcMAC21 had a higher RBC mean corpuscular volume (MCV) compared to euploid mice (Figure 7h; *p* < 0.05). There was a tendency for RBC mean corpuscular hemoglobin to be higher in TcMAC21 compared to euploid mice (Figure 7i; *p* = 0.06). Lastly, platelet count was similar between groups as well (Figure 7j; *p* > 0.05).

**FIGURE 8:**
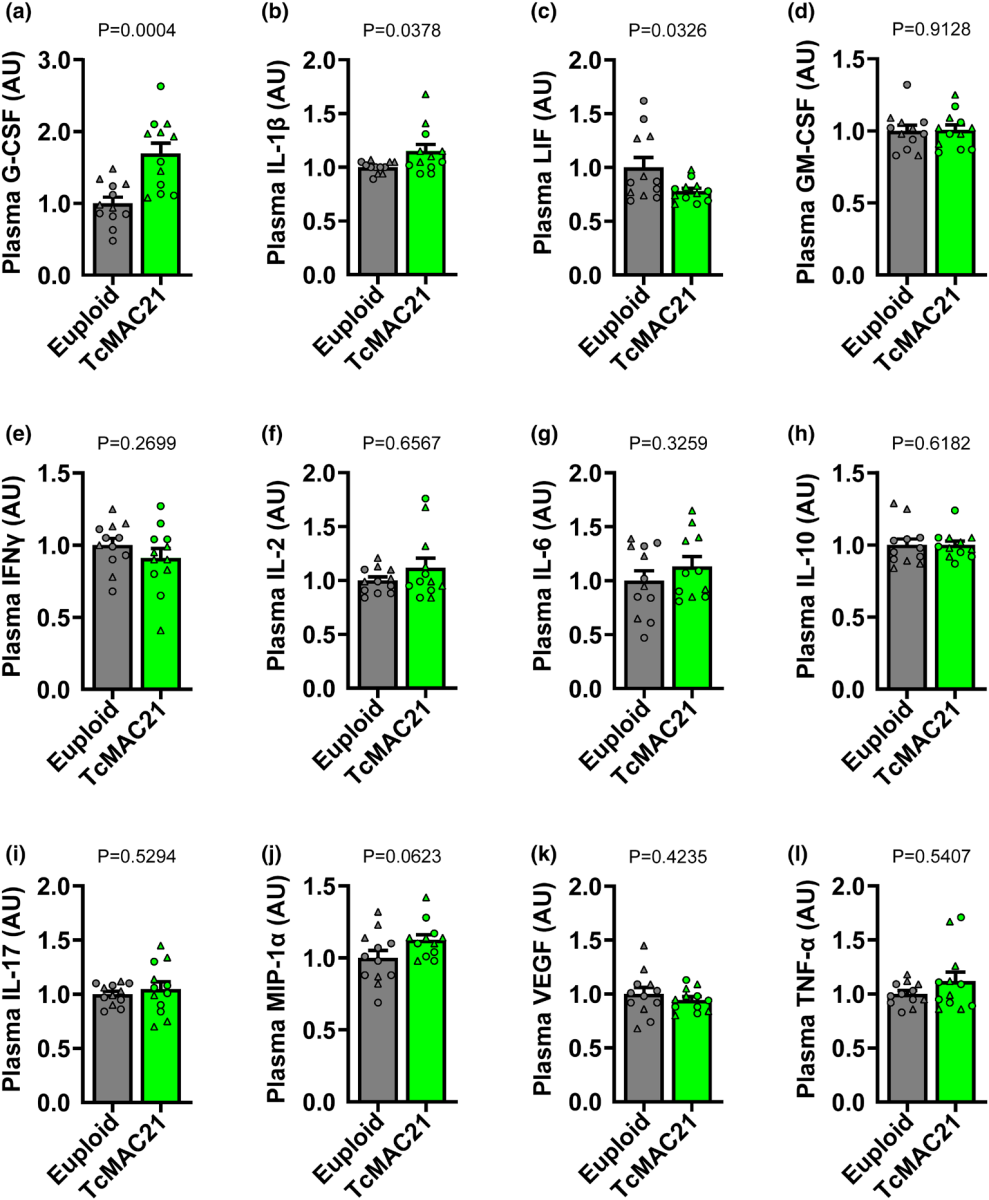
Group comparisons in euploid and TcMAC21 mice were analyzed using unpaired *t*‐test or Mann–Whitney *U* test to identify differences in plasma concentrations of G‐CSF (a; *n* = 12 mice/group), IL‐1β (b; *n* = 12 mice/group), LIF (c; *n* = 12 mice/group), GM‐CSF (d; *n* = 12 mice/group), IFNγ (e; *n* = 12 mice/group), IL‐2 (f; *n* = 12 mice/group), IL‐6 (g; *n* = 11–12 mice/group), IL‐10 (h; *n* = 12 mice/group), IL‐17 (i; *n* = 12 mice/group), MIP‐1α (j; *n* = 12 mice/group), VEGF (k; *n* = 12 mice/group), and TNF‐ α (l; *n* = 12 mice/group). Data are individual values (males = circles, females = triangles) and means ± SEM.

For the plasma cytokines/chemokines, TcMAC21 had higher levels of G‐CSF and IL‐1β (Figure 8a,b; *p* < 0.05 for all), and a lower level of LIF compared to euploid mice (Figure 8c; *p* < 0.05). Plasma levels of GM‐CSF, IFNγ, IL‐2, IL‐6, IL‐10, IL‐17, MIP‐1α, VEGF, and TNF‐a were similar between groups (Figure 8d–l; *p* > 0.05 for all). In addition, plasma levels of eotaxin, IL‐1α, IL‐4, IL‐3, IL‐5, IL‐7, IL‐9, IL‐12 (p40), IL‐12 (p70), IL‐13, LIX, IL‐15, IP‐10, KC, MCP‐1, MIP‐1β, M‐CSF, MIP‐2, MIG, and RANTES were similar between groups as well (Figure S2a–t; *p* > 0.05 for all).

There was a moderate, inverse relationship (*r* ^2^ = 0.11) between monocyte and glycocalyx thickness (Figure S3a; *p* < 0.05). There was a strong, inverse relationship (*r* ^2^ = 0.44) between plasma G‐CSF and glycocalyx thickness (Figure S3b; *p* < 0.05). There was no relationship (*r* ^2^ = 0.09) between plasma IL‐1β and glycocalyx thickness (Figure S3c; *p* > 0.05). There was a strong relationship (*r* ^2^ = 0.27) between plasma LIF and glycocalyx thickness (Figure S3d; *p* < 0.05).

## 4 DISCUSSION

In the present study, we observed lower systolic BP, lower aortic stiffness, and impaired endothelial function, indicated by a blunted flow‐mediated vasodilation, in TcMAC21 compared to euploid mice. Interestingly, we also observed a lower glycocalyx thickness in TcMAC21 mice, which may be a contributor to the blunted flow‐mediated vasodilation. Abnormal vascular physiology in TcMAC21 mice was also accompanied by elevated systemic inflammation, indicated by higher levels of circulating monocytes and pro‐inflammatory cytokines G‐CSF and IL‐1β and lower level of anti‐inflammatory cytokine LIF. In summary, these findings provide novel evidence that TcMAC21 mice exhibit abnormal vascular physiology that is similar to people with DS.

### 4.1 TcMAC21 mice exhibit abnormal vascular function and structure

Dyslipidemia, obesity, and lack of physical activity are more prevalent in people with DS than the general population (Roy‐Vallejo et al., 2020) and are strongly associated with elevated systolic BP and aortic stiffening (Cherfane et al., 2024; McEniery et al., 2010). However, people with DS typically exhibit low systolic BP and aortic stiffness (Roy‐Vallejo et al., 2020). In this study, we observed lower systolic BP and lower aortic stiffness in TcMAC21 mice, which suggests a similar phenotypical vasoprotection in people with DS and TcMAC21 mice. Although the mechanism responsible for this phenomenon is unclear, alterations in extracellular matrix proteins such as collagen deposition and elastin thinning and fragmentation are often associated with aortic stiffening (Pierce et al., 2022). In this study, we observed a similar aortic collagen content in euploid and TcMAC21 mice, but there was a higher elastin content in the aortas of TcMAC21 mice. It is likely that elevations in elastin content in the presence of similar aortic collagen may account for lower aortic stiffness and lower systolic BP in TcMAC21 mice (Agbaje, 2022; Weisbrod et al., 2013). Currently, there have been no studies that examined elastin or collagen content in the aorta of people with DS. Thus, future study is warranted in this area.

EDD is critical to endothelial health and is blunted prior to the development of CVD (Celermajer et al., 1994; Miller Jr. et al., 1998; Treasure et al., 1990; Widmer & Lerman, 2014). Despite lower systolic BP and aortic stiffness, TcMAC21 mice exhibited endothelial dysfunction. Studies that have examined vascular physiology in DS are scarce, although a previous study has shown that people with DS exhibit endothelial dysfunction as measured by changes in brachial blood flow to intraarterial acetylcholine infusion (Cappelli‐Bigazzi et al., 2004). Interestingly, we did not observe blunted carotid artery acetylcholine‐mediated vasodilation in TcMAC21 mice, although carotid artery flow‐mediated vasodilation was blunted in these mice. Surprisingly, there are no studies that have examined flow‐mediated vasodilation in people with DS, so there is very little known regarding the underlying mechanisms for endothelial dysfunction in DS. While we observed a discrepancy in measures of EDD in TcMAC21 mice, we did not observe any impairment in carotid artery vasoconstriction to phenylephrine or carotid artery vasodilation to sodium nitroprusside. Thus, blunted flow‐mediated vasodilation does not appear to be a consequence of blunted vascular contractility or impaired EID. An impairment in carotid artery flow‐mediated but not acetylcholine‐mediated vasodilation in TcMAC21 mice may suggest mechanisms underlying endothelial dysfunction involve mechanical but not pharmaceutical receptors/channels on the endothelium (Fang et al., 2019; Glassman et al., 2020). Clearly, more studies are warranted to examine vascular function in people with DS.

### 4.2 TcMAC21 mice exhibit altered Glycocalyx properties

To the best of our knowledge, this is the first study to investigate any aspect of glycocalyx properties in any model of DS. It is important to note that glycocalyx properties have never been studied in humans with DS either. The glycocalyx is known to modulate microvascular blood flow homogeneity and vascular permeability (Pries & Secomb, 2005; van den Berg et al., 2003). In this study, we observed that glycocalyx barrier function was not altered in TcMAC21 mice, as measured by PBR, but there was a lower glycocalyx thickness in the mesenteric microcirculation of these mice. The glycocalyx is known to mechanotransduce fluid shear stress from blood flow to endothelial cells through its major components such as heparan sulfate and hyaluronan (Pahakis et al., 2007; Yao et al., 2007), which stimulates the production of NO by endothelial NO synthase (eNOS) on the endothelial cells (Sriram et al., 2016). In this study, the lower glycocalyx thickness observed in TcMAC21 mice may have caused an impaired mechanotransduction to fluid shear stress from flow, which may have contributed to the blunted flow‐mediated vasodilation observed in these mice. Future study is warranted in humans to determine if glycocalyx properties are also altered in people with DS, as glycocalyx degradation is hypothesized to be one of the initial events for large artery dysfunction and is prevalent in patients with CVD (Machin et al., 2019).

### 4.3 Potential sources of endothelial dysfunction in TcMAC21 mice

Another potential mechanism by which trisomy 21 may cause blunted flow‐induced vasodilation is vascular inflammation, which is well‐established as one of the underlying causes for endothelial dysfunction and aortic stiffening (Donato et al., 2018). People with DS have a high prevalence of autoimmune disorders, indicated by increased systemic inflammation (Huggard et al., 2020). In this study, we observed a greater quantity of monocytes in the blood of TcMAC21 mice. An increased circulating monocytes may be detrimental to vascular health as they can be recruited to sites of inflammation in the vasculature, where they differentiate into macrophages, infiltrate into the vascular wall, and become the major source of vascular inflammation (Lesniewski et al., 2011; Ruder et al., 2023). Endothelial glycocalyx acts as a barrier to WBCs adhesion to endothelium (Lipowsky, 2012), and a reduced glycocalyx thickness may have facilitated the inflammatory process in the vasculature of these TcMAC21 mice. Furthermore, we also observed higher levels of circulating pro‐inflammatory cytokines IL‐1β and G‐CSF and anti‐inflammatory cytokine LIF in the plasma of TcMAC21 mice. Pro‐inflammatory cytokines IL‐1β and G‐CSF are produced by immune cells such as monocytes or directly by endothelial cells (Du et al., 2015; Hadadi et al., 2016; Takahashi et al., 1996). IL‐1β has been shown to dose‐dependently blunt EDD in isolated aorta through induced oxidative stress (Mukohda et al., 2016), and a higher level of IL‐1β has been observed in people with DS (Broers et al., 2012). On the contrary, although G‐CSF induces inflammation, it has direct beneficial effects on endothelial cells by upregulating eNOS transcription, expression, phosphorylation, and activation, leading to an increased production of NO (Park et al., 2008), which may have explained the preserved acetylcholine‐mediated vasodilation in TcMAC21 mice despite of systemic inflammation. On the other hand, anti‐inflammatory LIF suppresses the differentiation of T helper 17 cells, which can promote the repair of damaged epithelium (Banner et al., 1998; McGeachy et al., 2019), and deficit LIF activity has been observed in people with DS (Hahn et al., 1976). In general, systolic inflammation is detrimental to the glycocalyx (Yamaoka‐Tojo, 2020), as it can induce its shedding by degrading some of the key structures of glycocalyx such as heparan sulfate (Milusev et al., 2023). Indeed, we observed moderate to strong, inverse relationships between glycocalyx thickness and monocyte as well as pro‐inflammatory cytokine IL‐1β and G‐CSF in these mice. On the contrary, we observed a strong relationship between glycocalyx thickness and anti‐inflammatory cytokine LIF. Thus, inflammation‐induced glycocalyx shedding may have caused the blunted flow‐mediated vasodilation as observed in TcMAC21 mice (Florian et al., 2003). Nevertheless, a more precise evaluation of vascular inflammation such as macrophage infiltration may provide additional mechanistic insight (Shirai et al., 2015).

### 4.4 TcMAC21 mice exhibit altered metabolism

In the present study, TcMAC21 mice exhibited a smaller body mass and body stature, as indicated by shorter tibia length. In general, a smaller body mass in TcMAC21 mice was likely responsible for lower tissue masses for many internal organs and skeletal muscles. However, when expressed relative to body mass or tibia length, TcMAC21 mice exhibited a heavier internal organs, although skeletal muscles remained lighter, indicating a susceptibility for sarcopenia independent of body mass that also occurs in people with DS (Coelho‐Junior et al., 2019). Muscles with lower masses in TcMAC21 mice mainly contain type‐2 muscle fibers (e.g., quadricep, gastrocnemius, and plantaris), but the soleus muscle, which has a higher mass in TcMAC21 mice, predominantly contains type‐1 muscle fibers. This may suggest an altered muscular metabolism in these TcMAC21 mice as type‐1 muscle fibers contains higher mitochondrial content and rely on slow‐oxidative metabolism as the main energy source compared to type‐2 muscle fibers (Talbot & Maves, 2016). Furthermore, altered systemic metabolism, such as hyperactivity and hypermetabolism, has been observed in TcMAC21 mice from other study (Sarver et al., 2023a). Indeed, a smaller body stature, as observed in TcMAC21 mice, is associated with a higher metabolic rate per body mass (Holliday et al., 1967). Although glucose tolerance was similar between euploid and TcMAC21 mice, TcMAC21 mice exhibited a higher insulin sensitivity, which may account for the previously observed hypermetabolism in these mice (Sarver et al., 2023a). In this study, we also observed a higher blood flow in mesenteric circulation, which may indicate an enhanced perfusion in organs to match the hypermetabolism in these mice (Roy & Secomb, 2021). Furthermore, we observed a higher MCV in the RBCs of TcMAC21 mice that may indicate macrocytosis, which has also been reported in people with DS (Wachtel & Pueschel, 1991). Interestingly, it has been suggested that high MCV in DS is associated with altered RBC metabolism, such as intracellular accumulation of lactate, implying an alteration in metabolism at the cellular level (Culp‐Hill et al., 2017). Thus, TcMAC21 mice appear to have altered metabolism at whole‐body, muscular, and cellular levels. Although the observations on metabolic functions, such as increased insulin sensitivity in TcMAC21 mice from this study are consistent with the only other study using this mouse model (Sarver et al., 2023a), it is important to note that people with DS exhibit insulin resistance (Fonseca et al., 2005), which challenges the use of the TcMAC21 mouse model for metabolic disease in DS. Future studies are warranted to untangle the mechanisms behind this discrepancy, as altered metabolism mediates the development of pathological phenotypes, such as cognitive disorders in people with DS (Dierssen et al., 2020; Moreau et al., 2021).

### 4.5 Limitations

This study has its limitations. In this study, we used tail cuff to measure arterial systolic BP; while not the gold standard for measurement of BP in mice, this method has been validated versus direct invasive measurements performed using an arterial catheter (Feng et al., 2008). Nevertheless, a limitation of the tail cuff method is that measurements of diastolic and mean arterial pressure may be less accurate; thus, we did not use them. It should be noted that while systolic BP is lower in TcMAC21 compared to euploid mice, future studies are warranted to measure BP in this mouse model using BP telemetry in conscious mice. In the general population, aortic remodeling with aging includes stiffening of the aorta as well as increased lumen diameter and medial CSA (O'Rourke & Hashimoto, 2007; Wu et al., 2013). In this study, there was a tendency for TcMAC21 mice to have smaller aortic lumen diameter and medial CSA, but it is unclear whether smaller aortic lumen diameter and medial CSA directly contribute to lower aortic stiffness. Although aortic intima‐media thickness between newborns with or without DS was similar and ~1.2% of newborns with DS have congenital stenosis in the proximal thoracic aorta (Sarici et al., 2017; Vasconcelos et al., 2015), it is currently unknown if the aortic remodeling, such as an increase in the aortic lumen diameter and medial CSA, also happens in humans with DS as they age. In this study, ex vivo vascular function and glycocalyx properties were assessed in carotid arteries and mesenteric circulation, respectively. Future studies are warranted to assess ex vivo vascular function and glycocalyx properties within the same vascular bed and to use additional visualization tools such as electron microscopy and atomic force microscopy to establish the direct link between endothelial function and glycocalyx (Drost et al., 2023; Marsh & Waugh, 2013). This will also help precisely determine the exact steps/components, such as reduced eNOS expression, that are impaired in the machinery of blunted flow‐mediated vasodilation in these TcMAC21 mice (Power et al., 2024). We observed lower glycocalyx thickness but preserved glycocalyx barrier function in the mesenteric microcirculation of TcMAc21 mice. While glycocalyx barrier function has been used to infer glycocalyx thickness (Donati et al., 2013; Van Loo et al., 2023), and we have observed a relationship between these measurements (Machin et al., 2017; Zheng, Li, et al., 2022), these are not the same measurement. Indeed, one of the initial studies that measured glycocalyx properties found that hyaluronidase, which degrades hyaluronan in the glycocalyx, does not impact glycocalyx thickness but does worsen glycocalyx barrier function (Henry & Duling, 1999). Currently, it is unclear which aspect of the glycocalyx is depleted in TcMAC21 mice. Future studies are warranted to better understand the glycocalyx in animal models of DS, as well as in people with DS. It is important to note that, although our observations on the metabolism of these TcMAC21 mice, such as higher insulin sensitivity, align with the only other study assessed metabolic function in these mice (Sarver et al., 2023b), people with DS are at an increased risk for insulin resistance (Dierssen et al., 2020). This discrepancy is possible due to a lack of physical activity and the higher prevalence of overweight and obesity in people with DS (Oreskovic et al., 2020; Real de Asua et al., 2014), which contributes to the development of insulin resistance (Kahn & Flier, 2000), while TcMAC21 mice exhibit hyperactivity with small body mass (Sarver et al., 2023b). Nevertheless, this discrepancy in metabolism patterns between TcMAC21 mice and people with DS needs to be thoroughly examined by future studies, as it stands as an impediment for the generalization of this mouse model of DS for future translational studies, as metabolism impacts the entire body system. Lastly, although this study included both sexes of mice, it was underpowered to detect sex‐related differences due to a small sample size of female TcMAC21 mice. We speculate that female TcMAC21 mice exhibit a higher prevalence of congenital heart disease, as observed in humans with DS (Diogenes et al., 2017), which may have caused a lower survival rate of female pups. Future studies are warranted to confirm this speculation and explore the potential sex differences in the vascular physiology of TcMAC21 mice.

## 5 CONCLUSIONS

In the present study, we observed altered body mass, stature, and composition, enhanced metabolic function, and abnormal vascular physiology in male and female TcMAC21 compared to euploid (i.e., control) mice. Specifically, TcMAC21 mice exhibited a smaller body mass and tibia length, a higher composition of slow‐oxidative muscle, and a higher insulin sensitivity. Despite systolic BP and arterial stiffness being lower in TcMAC21 mice, they exhibited endothelial dysfunction, indicated by blunted carotid artery flow‐mediated vasodilation, which may have been due to a reduced glycocalyx thickness, as observed in the mesenteric microcirculation. Another potential contributor for the blunted flow‐mediated vasodilation in these mice is systemic inflammation, indicated by higher levels of circulating monocytes and pro‐inflammatory cytokines G‐CSF and IL‐1β and a lower level of anti‐inflammatory cytokine LIF. Although the precise mechanisms for these physiological alterations in TcMAC21 mice are not entirely clear, these data provide preliminary and direct support for the use of the TcMAC21 mouse model to study the abnormal vascular physiology in people with DS. Future studies are warranted to elucidate the mechanisms behind the abnormal vascular physiology in TcMAC21 mice, as it provides translational insight into the altered paradigm of vascular function profile in the DS population, which could have enormous implications for CVD management in people with DS and boost the development of novel therapeutic targets in CVD prevention for the general population.

## FUNDING INFORMATION

This work was funded in part by a grant from the National Institutes of Health (R00 AT010017).

## CONFLICT OF INTEREST STATEMENT

No conflicts of interest, financial or otherwise, are declared by the authors.

## Supporting information


Appendix S1.

## Data Availability

The data that support the findings of this study are available from the corresponding author upon reasonable request.
