# Supplementary material for: TcMAC21 mouse model recapitulates abnormal vascular physiology observed in humans with Down syndrome
Source: Physiol Rep. 2025 Jun 6;13(11):e70384. doi: 10.14814/phy2.70384 (PMC12141928; doi:10.14814/phy2.70384)
Supplement: Supplementary file 1 — Appendix S1. [file PHY2-13-e70384-s001.docx]

Supplemental Information for

**TcMAC21 Mouse Model Recapitulates Abnormal Vascular Physiology in Humans with Down Syndrome**

Contact e-mail: dmachin@salud.unm.edu

**
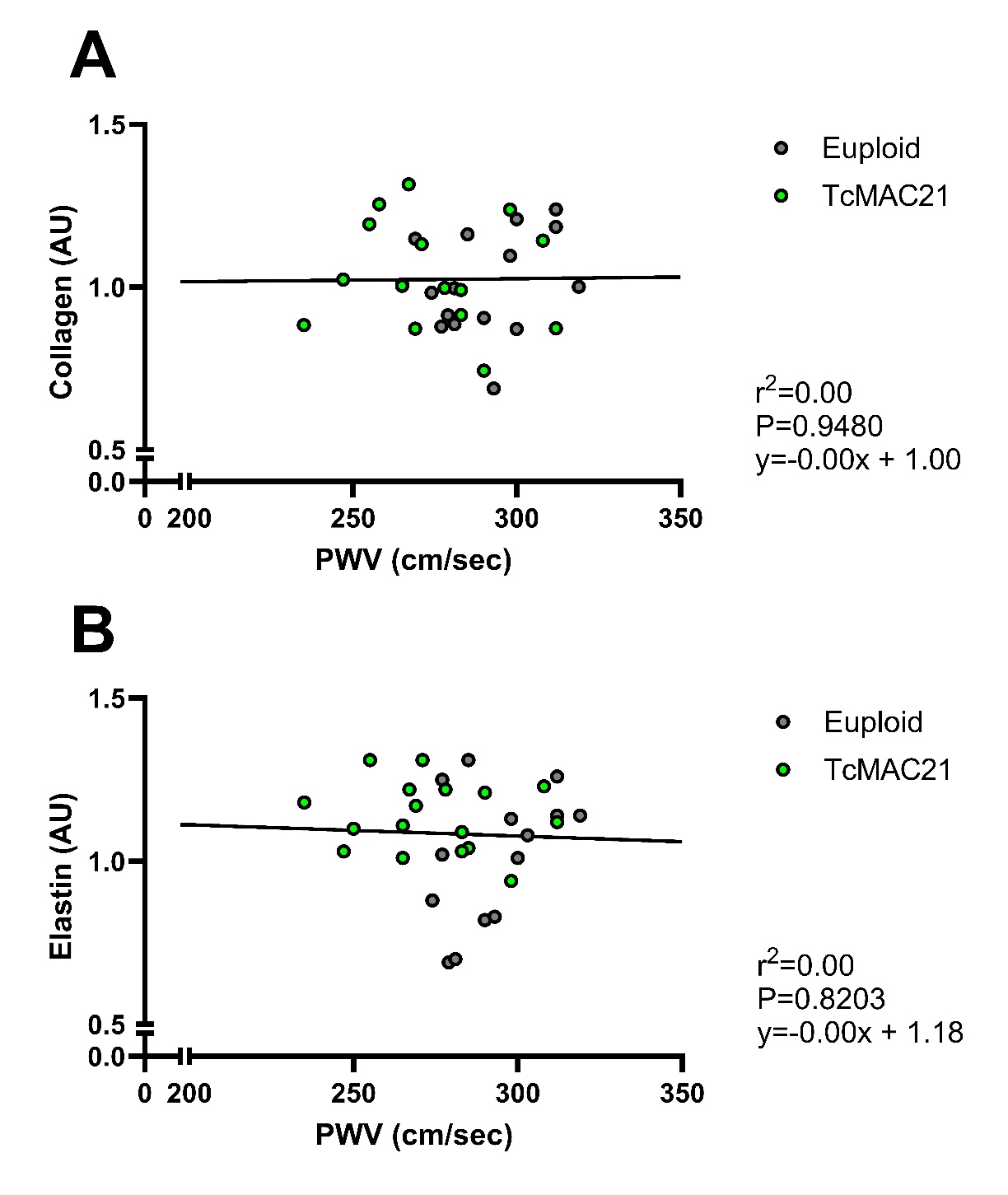
Figure S1**

**Figure S1.** Bivariate linear regression analysis was used to determine the relationship between aortic pulse wave velocity (**PWV**) and aortic content of collagen (**A**) and elastin (**B**) in euploid and TcMAC21 mice.

**Figure S2**

**
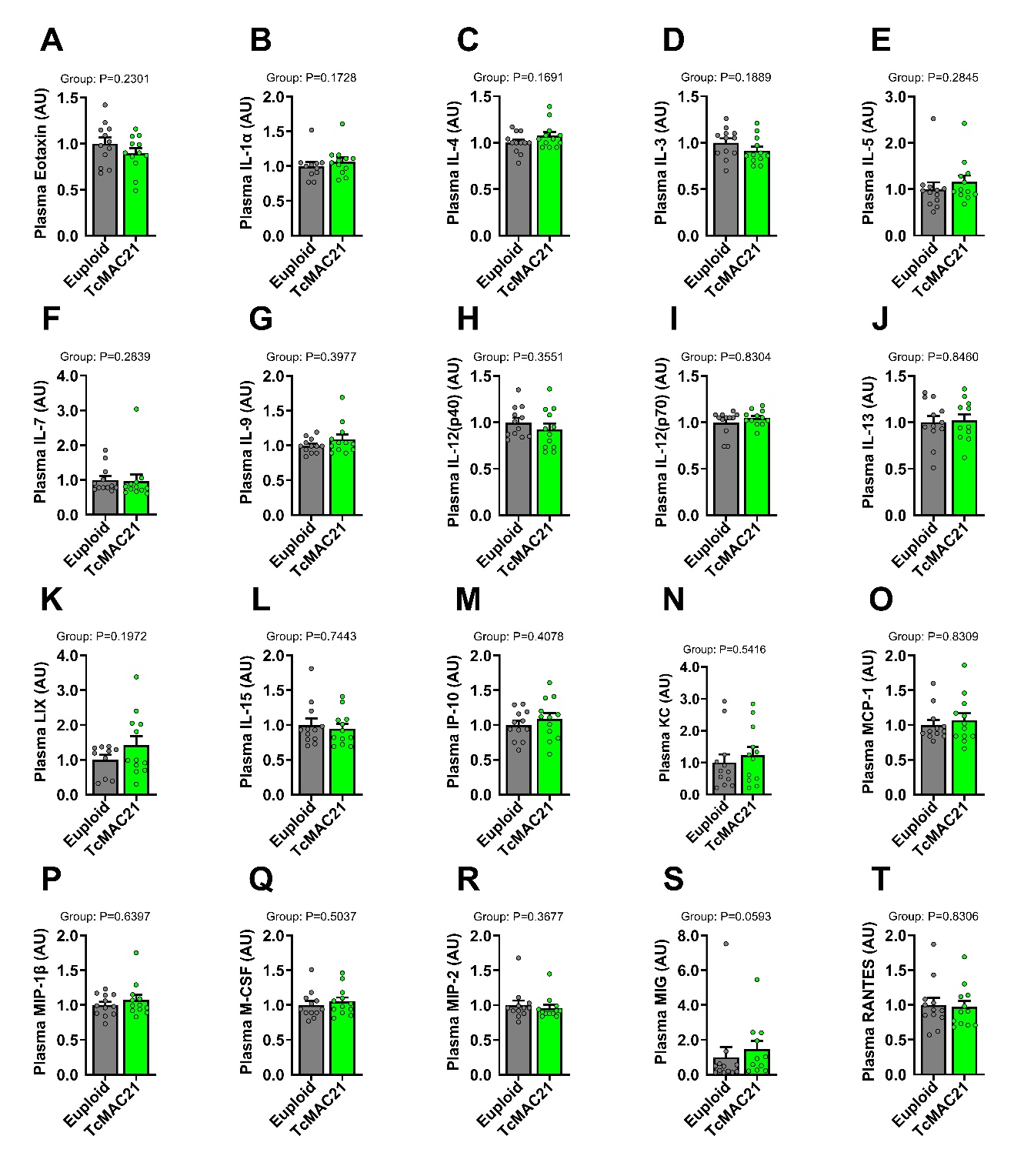
**

**Figure S2.** Group comparisons in euploid and TcMAC21 mice were analyzed using unpaired t test or Mann-Whitney U test to identify differences in plasma concentrations of eotaxin (**A**), IL-1α (**B**), IL-4 (**C**), IL-3 (**D**), IL-5 (**E**), IL-7 (**F**), IL-9 (**G**), IL-12 (p40; **H**), IL-12 (p70; **I**), IL-13 (**J**), LIX (**K**), IL-15 (**L**), IP-10 (**M**), KC (**N**), MCP-1 (**O**), MIP-1β (**P**), M-CSF (**Q**), MIP-2 (**R**), MIG (**S**), and RANTES (**T**). Data are individual values and means±SEM. n = 10-12 mice/group.

**
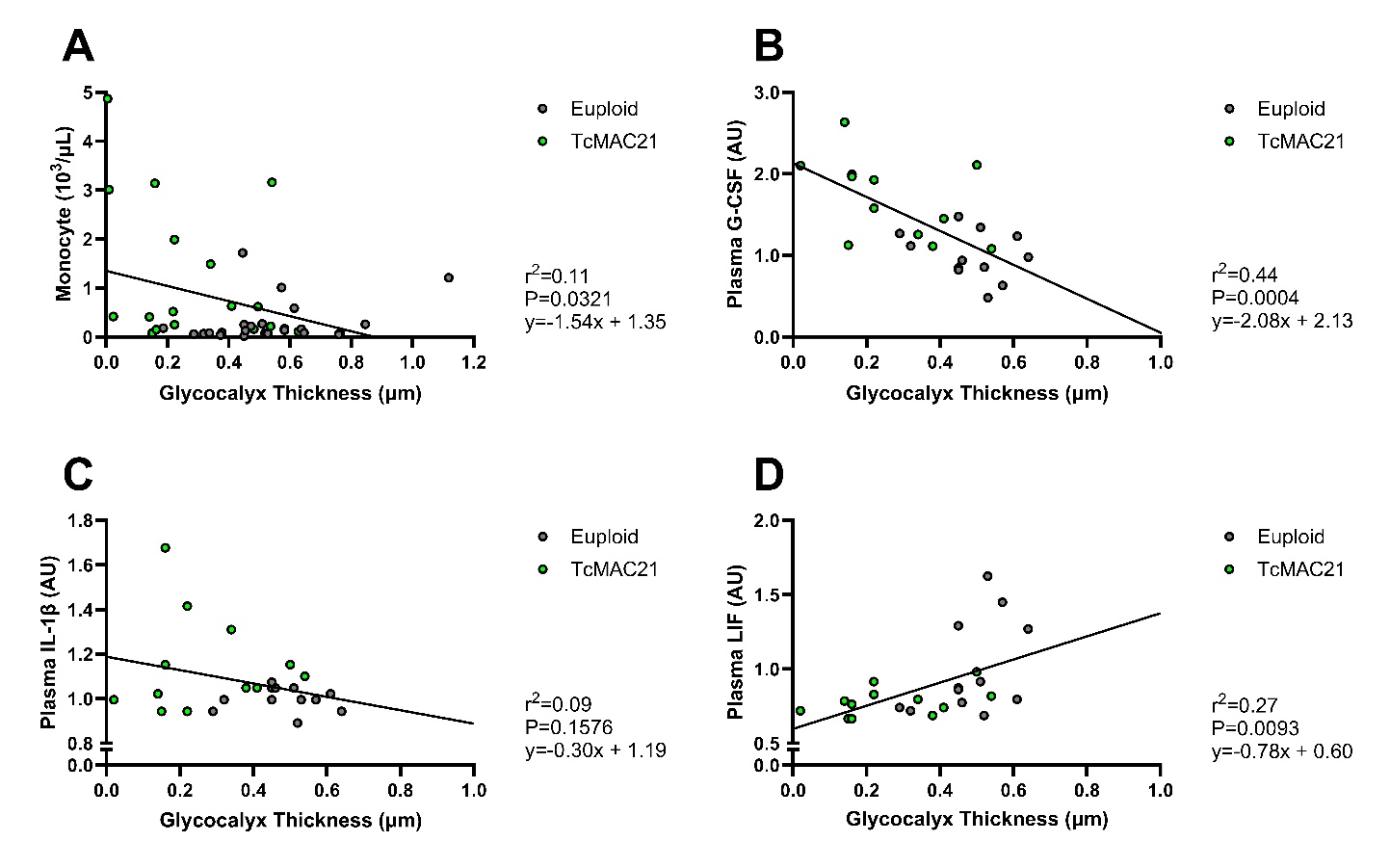
Figure S3**

**Figure S3.** Bivariate linear regression analysis was used to determine the relationship between glycocalyx thickness and monocyte (**A**), glycocalyx thickness and plasma G-CSF (**B**), glycocalyx thickness and plasma IL-1β (**C**), and glycocalyx thickness and plasma LIF (**D**) in euploid and TcMAC21 mice.

**Table S1. Animal Characteristics**

|  | **Euploid** | **TcMAC21** | **Group** |
| --- | --- | --- | --- |
| Heart/body mass, mg/g | 4.5±0.1 | 5.1±0.1* | <0.0001 |
| Heart/tibia length, mg/mm | 0.25±0.01 | 0.29±0.01* | 0.0002 |
| Liver/body mass, mg/g | 48.7±1.2 | 58.2±1.4* | <0.0001 |
| Liver/tibia length, mg/mm | 2.70±0.07 | 3.31±0.09* | <0.0001 |
| Spleen/body mass, mg/g | 3.0±0.2 | 3.4±0.2 | 0.1176 |
| Spleen/tibia length, mg/mm | 0.17±0.01 | 0.19±0.01 | 0.0643 |
| Quadricep/body mass, mg/g | 6.8±0.2 | 4.4±0.1* | <0.0001 |
| Quadricep/tibia length, mg/mm | 0.38±0.01 | 0.25±0.01* | <0.0001 |
| Gastrocnemius/body mass, mg/g | 4.7±0.1 | 3.0±0.1* | <0.0001 |
| Gastrocnemius/tibia length, mg/mm | 0.26±0.01 | 0.17±0.01* | <0.0001 |
| Soleus/body mass, mg/g | 0.3±0.0 | 0.4±0.0* | <0.0001 |
| Soleus/tibia length, mg/mm | 0.01±0.00 | 0.02±0.00* | <0.0001 |
| Plantaris/body mass, mg/g | 0.7±0.0 | 0.6±0.0* | 0.0066 |
| Plantaris/tibia length, mg/mm | 0.04±0.00 | 0.03±0.00* | 0.0075 |
| Kidney/body mass, mg/g | 7.2±0.1 | 8.2±0.2* | 0.0010 |
| Kidney/tibia length, mg/mm | 0.40±0.01 | 0.47±0.02* | 0.0014 |
| VAT/body mass, mg/g | 13.9±2.0 | 10.0±1.1 | 0.1798 |
| VAT/tibia length, mg/m | 0.77±0.11 | 0.56±0.06 | 0.2229 |
| SAT/body mass, mg/g | 6.7±0.6 | 5.9±0.4 | 0.3394 |
| SAT/tibia length, mg/m | 0.37±0.03 | 0.34±0.02 | 0.4826 |

Group comparisons in euploid and TcMAC21 mice were analyzed using unpaired t test or Mann-Whitney U test to identify differences in animal characteristics. VAT, visceral adipose tissue; SAT, subcutaneous adipose tissue. *P<0.05 vs. euploid. Data are individual values and means±SEM.

**Table S2. Sex-Specific Animal Characteristics**

|  | **Euploid** | | **TcMAC21** | | **Group** | **Sex** | | **Interaction** | |  |
| --- | --- | --- | --- | --- | --- | --- | --- | --- | --- | --- |
|  | **M** | **F** | **M** | **F** |  | |  | |  | |
| Male/Female | 11 | 11 | 12 | 7 |  | |  | |  | |
| Age, mo | 4.2±0.1 | 4.5±0.1 | 4.4±0.1 | 4.4±0.1 | 0.1182 | | 0.9819 | | 0.9819 | |
| Body mass, g | 30.8±0.9 | 25.8±0.9† | 22.3±0.8* | 19.4±1.0*† | <0.0001 | | <0.0001 | | 0.2496 | |
| Tibia length, mm | 17.9±0.2 | 18.1±0.1 | 17.4±0.2 | 18.0±0.2 | 0.0939 | | 0.0756 | | 0.4366 | |
| Heart, mg | 140.9±4.0 | 113.2±1.2† | 114.8±4.0* | 97.4±5.4*† | <0.0001 | | <0.0001 | | 0.1937 | |
| Heart/body mass, mg/g | 4.6±0.1 | 4.4±0.2* | 5.2±0.1* | 5.0±0.1* | <0.0001 | | 0.2992 | | 0.9647 | |
| Heart/tibia length, mg/mm | 0.26±0.01 | 0.25±0.1* | 0.30±0.01* | 0.28±0.01* | 0.0004 | | 0.060 | | 0.9625 | |
| Liver, mg | 1410±55 | 1318±46 | 1304±42 | 1096±48*† | 0.0020 | | 0.0042 | | 0.2425 | |
| Liver/body mass, mg/g | 45.9±1.8 | 51.3±1.3† | 58.9±1.9* | 57±2.1* | <0.0001 | | 0.3440 | | 0.0480 | |
| Liver/tibia length, mg/mm | 2.56±0.01 | 2.83±0.08* | 3.39±0.12* | 3.17±0.13* | <0.0001 | | 0.7897 | | 0.0380 | |
| Spleen, mg | 76.8±3.1 | 87.9±7.4 | 69.9±3.8 | 75.3±5.9 | 0.0823 | | 0.1399 | | 0.6022 | |
| Spleen/body mass, mg/g | 2.5±0.1 | 3.4±0.3† | 3.2±0.2* | 3.9±0.2 | 0.0308 | | 0.0035 | | 0.6436 | |
| Spleen/tibia length, mg/mm | 0.14±0.01 | 0.19±0.02† | 0.18±0.01* | 0.22±0.01 | 0.0193 | | 0.0051 | | 0.5927 | |
| Quadricep, mg | 207±9 | 176±7† | 100±6* | 82±4* | <0.0001 | | 0.0026 | | 0.3693 | |
| Quadricep/body mass, mg/g | 6.7±0.3 | 6.9±0.4* | 4.5±0.2* | 4.3±0.1* | <0.0001 | | 0.9998 | | 0.4976 | |
| Quadricep/tibia length, mg/mm | 0.38±0.02 | 0.38±0.02 | 0.26±0.01* | 0.24±0.01* | <0.0001 | | 0.6874 | | 0.4868 | |
| Gastrocnemius, mg | 143±4 | 122±6† | 71±5* | 54±4*† | <0.0001 | | 0.0009 | | 0.7321 | |
| Gastrocnemius/body mass, mg/g | 4.7±0.2 | 4.8±0.2* | 3.2±0.2* | 2.8±0.2* | <0.0001 | | 0.4231 | | 0.2486 | |
| Gastrocnemius/tibia length, mg/mm | 0.26±0.01 | 0.26±0.01* | 0.18±0.01* | 0.16±0.01* | <0.0001 | | 0.3787 | | 0.2751 | |
| Soleus, mg | 7.9±0.4 | 7.0±0.5 | 8.8±0.4 | 9.0±1.4* | 0.0368 | | 0.6240 | | 0.3875 | |
| Soleus/body mass, mg/g | 0.3±0.0 | 0.3±0.0* | 0.4±0.0* | 0.5±0.0* | <0.0001 | | 0.4323 | | 0.3513 | |
| Soleus/tibia length, mg/mm | 0.01±0.00 | 0.01±0.00* | 0.02±0.00* | 0.03±0.01* | <0.0001 | | 0.2958 | | 0.2334 | |
| Plantaris, mg | 19.1±0.9 | 12.2±0.6† | 19.1±0.9* | 12.2±0.6* | <0.0001 | | 0.0093 | | 0.1369 | |
| Plantaris/body mass, mg/g | 0.7±0.1 | 0.7±0.0* | 0.6±0.0* | 0.6±0.0 | 0.0050 | | 0.6325 | | 0.3362 | |
| Plantaris/tibia length, mg/mm | 0.04±0.00 | 0.04±0.00* | 0.03±0.00* | 0.03±0.00 | 0.0053 | | 0.3176 | | 0.3666 | |
| Kidney, mg | 229±9 | 179±4† | 191±8* | 143±5*† | <0.0001 | | <0.0001 | | 0.8990 | |
| Kidney/body mass, mg/g | 7.5±0.3 | 7.0±0.1 | 8.6±0.3* | 7.4±0.3† | 0.0024 | | 0.0026 | | 0.1831 | |
| Kidney/tibia length, mg/mm | 0.42±0.02 | 0.39±0.01* | 0.50±0.02* | 0.41±0.02† | 0.0033 | | 0.0023 | | 0.1390 | |
| VAT, mg | 534±127 | 312±76 | 211±23* | 229±63 | 0.0181 | | 0.2227 | | 0.1529 | |
| VAT/body mass, mg/g | 16.6±3.3 | 11.4±1.1 | 9.2±0.9* | 11.2±2.7 | 0.1164 | | 0.5009 | | 0.1324 | |

Group comparisons in male and female euploid and TcMAC21 mice were analyzed using two-way ANOVA with Holm-Šídák post hoc test to identify differences in animal characteristics. VAT, visceral adipose tissue; SAT, subcutaneous adipose tissue. *P<0.05 vs. euploid within the sex. †P<0.05 vs. male within the group. Data are individual values and means±SEM.
